# Depolymerization of biorefinery lignin by improved laccases of the white‐rot fungus *Obba rivulosa*

**DOI:** 10.1111/1751-7915.13896

**Published:** 2021-07-26

**Authors:** Janne Wallenius, Jussi Kontro, Christina Lyra, Jaana Kuuskeri, Xing Wan, Mika A. Kähkönen, Irshad Baig, Paul C. J. Kamer, Jussi Sipilä, Miia R. Mäkelä, Paula Nousiainen, Kristiina Hildén

**Affiliations:** ^1^ Fungal Genetics and Biotechnology Department of Microbiology University of Helsinki University of Helsinki Biocenter 1 PO Box 56 Viikinkaari 9 Helsinki FI‐00014 Finland; ^2^ Department of Chemistry University of Helsinki P.O. Box 55 A. I. Virtasen Aukio 1 Helsinki FI‐00014 Finland; ^3^ EaStCHEM School of Chemistry University of St Andrews Fife UK; ^4^ Present address: Department of Organic Synthesis and Process Chemistry CSIR‐Indian Institute of Chemical Technology 500 007 Tarnaka, Hyderabad India

## Abstract

Fungal laccases are attracting enzymes for sustainable valorization of biorefinery lignins. To improve the lignin oxidation capacity of two previously characterized laccase isoenzymes from the white‐rot fungus *Obba rivulosa*, we mutated their substrate‐binding site at T1. As a result, the pH optimum of the recombinantly produced laccase variant r*Or*Lcc2‐D206N shifted by three units towards neutral pH. *O. rivulosa* laccase variants with redox mediators oxidized both the dimeric lignin model compound and biorefinery poplar lignin. Significant structural changes, such as selective benzylic α‐oxidation, were detected by nuclear magnetic resonance analysis, although no polymerization of lignin was observed by gel permeation chromatography. This suggests that especially r*Or*Lcc2‐D206N is a promising candidate for lignin‐related applications.

## Graphical abstract

Site‐directed mutagenesis shifted laccase pH range towards neutral by three pH units. LMS showed significant structural modifications of biorefinery lignin. Selective benzylic oxidation of lignin by LMS was observed.

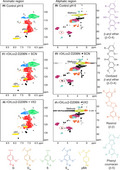

## Introduction

Lignocellulosic biorefinery concept utilizes various plant biomass resources for the production of biofuels and biochemicals aiming to zero‐waste model. These biorefineries, however, generate side‐streams that are difficult to convert into value‐added products. Lignin‐containing side‐stream fraction is the most abundant and challenging in downstream processing, due to the extreme recalcitrance and structural heterogeneity (Schutyser *et al*., 2018). The laccase‐catalysed treatment has a great potential to sustainable transform of lignin‐rich side‐streams into value‐added small molecules and other high value products (Roth and Spiess, 2015).

Laccases (EC 1.10.3.2) are copper (Cu)‐containing oxidoreductase enzymes commonly present in wood‐degrading fungal species. Typically, fungal laccases have four Cu atoms in their catalytic centre (Giardina *et al*., 2010). Substrate‐binding Type 1 (T1) Cu is located close to the protein surface, and a cluster of Type 2 (T2) Cu and two Type 3 (T3) Cu atoms are buried inside the protein. Molecular oxygen is reduced to water at the T2/T3 site by the electrons transferred from the T1 site through highly conserved His–Cys–His tripeptide. The reduction of T1 Cu by the substrate is the rate‐limiting step of the reaction (Giardina *et al*., 2010; Mehra and Kepp, 2019).

Laccases have a wide substrate range, ideal substrates being phenolic compounds such as dihydroxyphenols. Structural units that have higher redox potential, such as non‐phenolic lignin structures, are recalcitrant to direct laccase oxidation. However, in the presence of redox mediators, laccases are able to catalyse oxidation of non‐phenolic structures and cleavage of inter‐unit linkages in lignin (Munk *et al*., 2015). Redox mediators are small, diffusible electron carriers, which can access the T1 Cu‐active site and act as electron shuttles between the oxidant and the target substrate (Cañas and Camarero, 2010). The substrate repertoire of laccases can be expanded by laccase‐mediator systems (LMS), and several different oxidation mechanisms have been described for diverse mediators (Roth and Spiess, 2015).

As laccases require only molecular oxygen for catalysis, they are prominent candidates for various biotechnological applications (Zerva *et al*., 2019). Besides having potential in lignocellulosic biomass processing, laccases can be utilized in polymer grafting, organic synthesis and polymerization, and processing of food and beverages (Rodríguez Couto and Toca‐Herrera, 2006), as well as in biosensors for phenolic compounds. In addition, laccases are promising biocatalysts for bioremediation of various xenobiotics, such as dyes and pharmaceutical compounds (Mäkelä *et al*., 2020).

White‐rot fungal laccases have high redox potential at the T1 active site, which provides the benefit for a wider range of oxidizable substrates than bacterial and plant laccases. They are even able to modify and depolymerize lignin (Sitarz *et al*., 2016). However, most so far characterized white‐rot fungal laccases function at the acidic environment, where lignin is insoluble, thus hampering their use in applications related to lignin depolymerization. For industrial purposes, ideal laccases would possess features such as high redox potential, robustness in wide pH and temperature range, and tolerance towards free radicals and organic solvents (Sitarz *et al*., 2016).

We conducted site‐directed mutagenesis on two previously characterized high‐redox potential laccase isoenzymes Lcc1 and Lcc2 of the white‐rot fungus *Obba rivulosa* (Hildén *et al*., 2013; Kontro *et al*., 2020), to improve their narrow acidic pH range towards neutral conditions and thus their efficiency for applications related to lignin depolymerization. The substrate‐binding site of *O. rivulosa* Lcc1 and Lcc2 is highly conserved; however, the size of the substrate‐binding cavity and the surface electrostatic charge vary (Hildén *et al*., 2013). Individual amino acid residues in the vicinity of the T1 substrate‐binding site have been shown to be important with respect to pH dependence of laccase activity (Bertrand *et al*., 2002; Giardina *et al*., 2010). Therefore, we hypothesized that by modifying the selected conserved amino acids at the T1 site, the pH profile of *O. rivulosa* laccases could be shifted towards alkaline pH region, where phenolic substrates have lower redox potential with better solubility and with no expense to the original enzyme redox potential. The laccase variants were heterologously expressed in *Pichia pastoris* and characterized, and their ability to oxidize lignin model compounds in the presence of synthetic and natural mediators was assessed. We examined the applicability of the laccase variants in combination with the mediators to depolymerize biorefinery lignin and identified the main modifications in the lignin structure by gel permeation chromatography (GPC), infrared spectroscopy (IR) and nuclear magnetic resonance spectroscopy (NMR).

## Results and discussion

### Characterization of r*Or*Lcc1‐D208N and r*Or*Lcc2‐D206N by experimental and *in silico* analyses

**Fig. 1:**
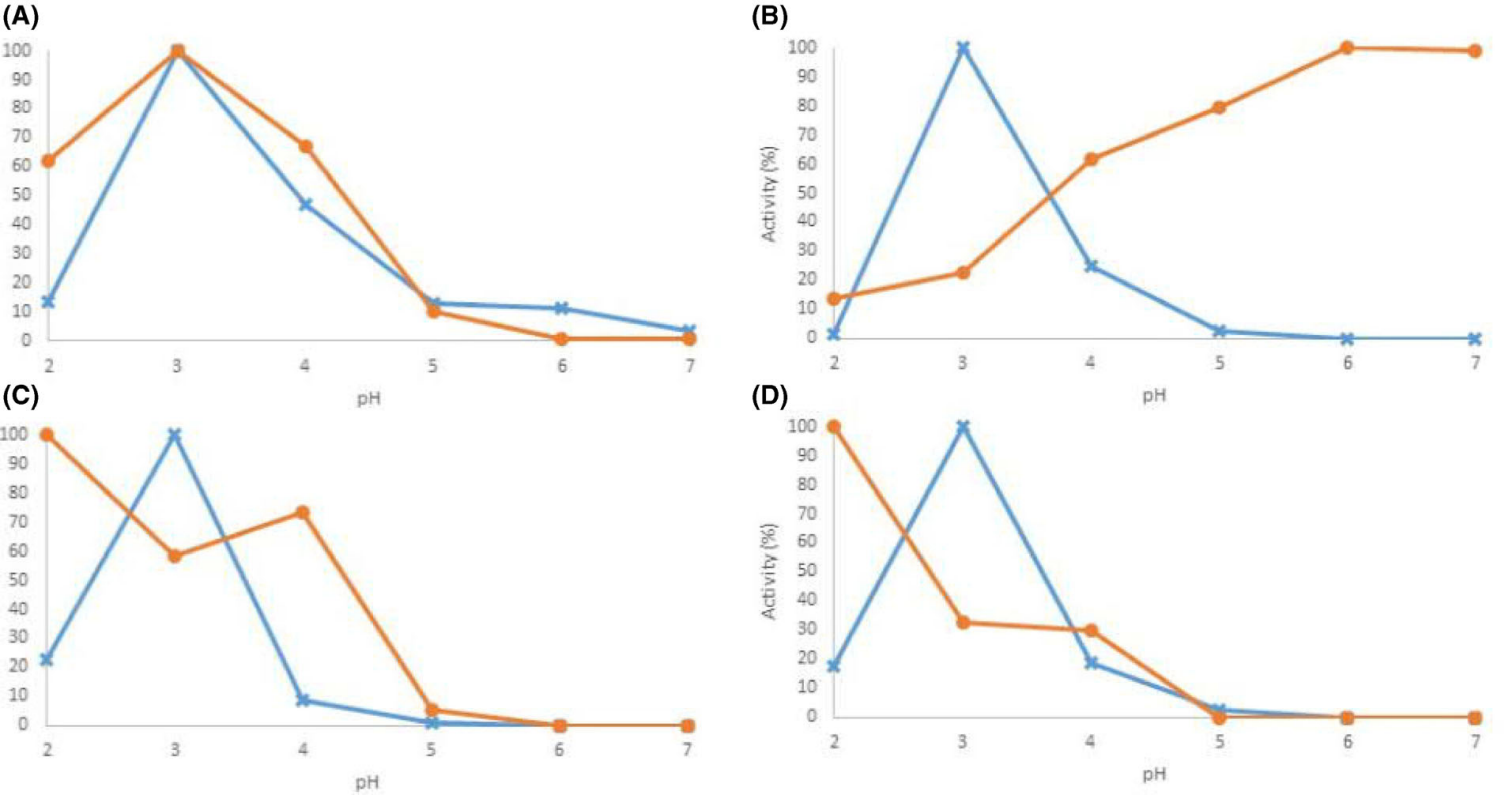
Residual activity of parental (blue cross) and variant *O. rivulosa* laccases (orange circle) towards 2,6‐DMP and ABTS (A) r*Or*Lcc1 and r*Or*Lcc1‐D208N with 2,6‐DMP; (B) r*Or*Lcc2 and r*Or*Lcc2‐D206N with 2,6‐DMP; (C) r*Or*Lcc1 and r*Or*Lcc1‐D208N with ABTS. (D) r*Or*Lcc2 and r*Or*Lcc2‐D206N with ABTS. The activities are normalized to the maximum activity. The measurements were carried out as triplicates, and averages are plotted. The standard deviation did not exceed 1%.

Two previously characterized high‐redox potential laccases of the white‐rot fungus *O*. *rivulosa*, which have acidic pH optima (pH 3.5) and extremely narrow working range in their catalysis towards phenolic substrates (Hildén *et al*., 2013), were selected as targets for site‐directed mutagenesis. Hydrophilic Asp residues (Asp208 of *Or*Lcc1 and Asp206 of *Or*Lcc2) in the vicinity of the active site T1 were replaced with positively charged Asn. As a result, the pH optimum of r*Or*Lcc2‐D206N (pH 6.0‐7.0) shifted three units towards alkaline pH compared with the parental laccase (pH 3.5) when 2,6‐DMP was used as a substrate (Fig. 1 and Fig. S1, Table S1). Similarily with our results, replacement of Asp206 to Asn in a basidiomycete laccase has resulted in 1.4‐2 unit increase in the optimum pH value for 2,6‐DMP (Madzak *et al*., 2006; Mate *et al*., 2013). In addition, plant laccases with a conserved Asn206 residue are active at neutral to alkaline pH conditions (Madzak *et al*., 2006). For r*Or*Lcc1‐D208N, the working pH range did not change. For both variants, the highest activity towards ABTS was detected at pH 2.0, whereas only low residual activity (approx. 20%) was measured for the parental laccases.

The interactions between 2,6‐DMP and T1 active sites of r*Or*Lcc1‐D208N and r*Or*Lcc2‐D206N were investigated by molecular docking, followed by MD simulations and subsequentially binding affinity analyses with MMGBSA over MD trajectories (Table S2, Fig. S1). The averaged binding affinities were higher for the both variants than the parental laccases. The asparagine mutation of *Or*Lcc2‐D206N seemed to stabilize 2,6‐DMP at the T1 active site, which was also experimentally observed with higher binding affinities than *Or*Lcc2 (Fig. S3). The correlation between low *K_m_* values and the high binding affinities in laccases has been shown previously with *Trametes versicolor* laccase (Mehra *et al*., 2018). In contrast to our results, *T. versicolor* D206N laccase variant showed reduced 2,6‐DMP activity due to the possible premature movement of 2,6‐DMP from the active site.

Recombinant *O. rivulosa* laccases were purified to homogeneity in the two‐step purification process resulting in 8.6% and 1.5% yield for r*Or*Lcc1‐D208N and r*Or*Lcc2‐D206N, respectively (Fig. S1, Table S1). Both laccase variants appeared as single bands in the SDS–PAGE. The catalytic properties of the laccases were determined by using 2,6‐DMP as a substrate with the purified laccase fractions (Table 1). The catalytic efficiency of r*Or*Lcc2‐D206N was significantly higher (2947 mM^−1^ s^−1^) than that of r*Or*Lcc1‐D208N (12 mM^−1^ s^−1^) and almost 20‐fold higher than the parental r*Or*Lcc2 (160 mM^−1^ s^−1^) at the optimal pH conditions.

**Table 1:** Kinetic constants for parental and variant *O. rivulosa* laccases towards 2,6‐DMP at their optimal pH.

Enzyme	r*Or*Lcc1^a^	r*Or*Lcc1‐D208N	r*Or*Lcc2‐L497V^a^	r*Or*Lcc2‐D206N
*K*_m_ (mM)	0.039 ± 0.00	3.668 ± 0.27	0.206 ± 0.00	0.038 ± 0.00
*V*_max_ (μkat l^‐1^)	7 ± 0.01	3.6 ± 0.17	57 ± 0.17	2.5 ± 0.18
*k*_cat_ (s^‐1^)	5 ± 0.01	44	34 ± 0.1	112
*k*_cat_/*K*_m_ (mM^−1^ s^−1^)	130	12	160	2947

The measurements were carried out as triplicates. The error range is given as 95% confidence interval.

^a^ The values are from Hildén *et al*. (2013).

**Fig. 2:**
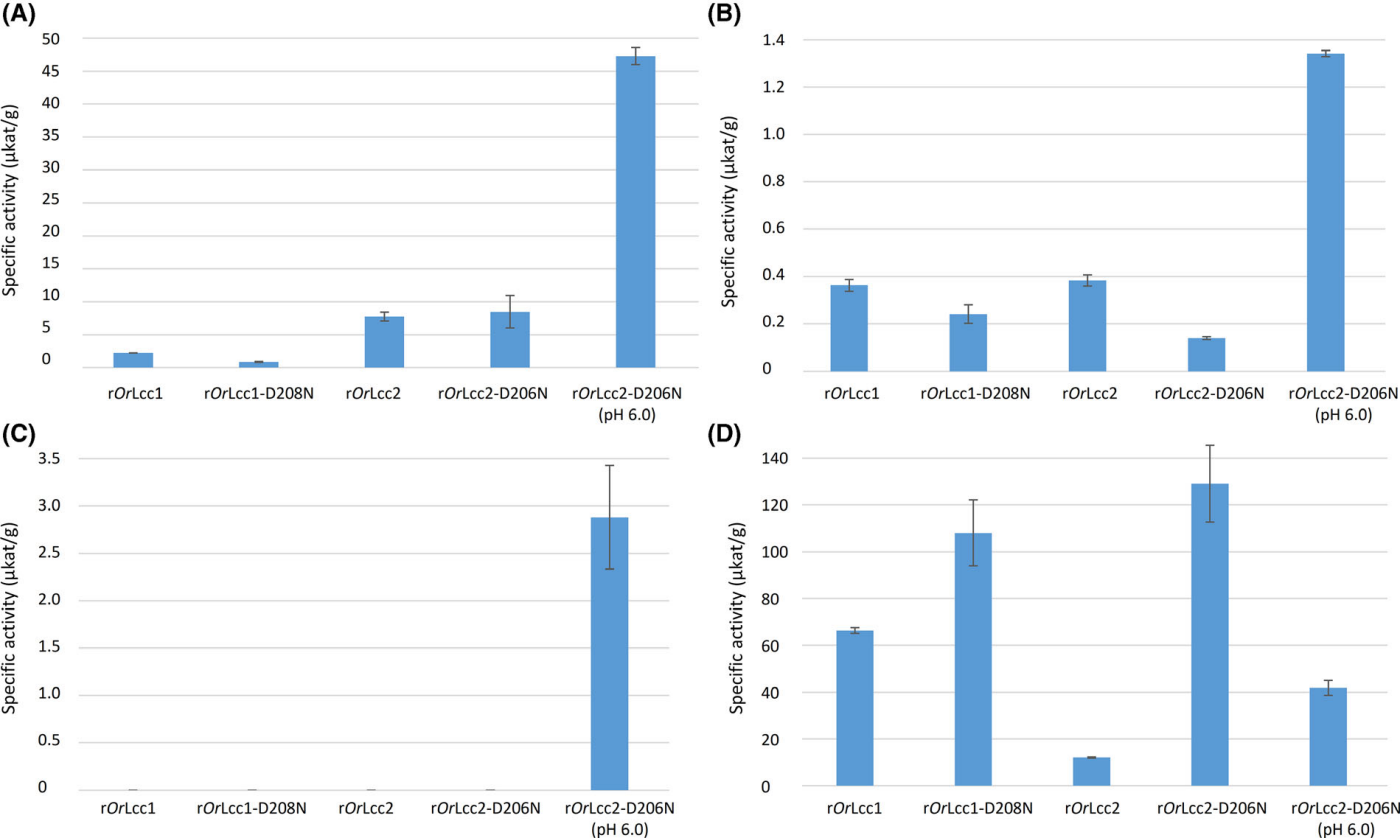
Substrate specificities of *O. rivulosa* parental laccases and variants. The activities of crude extracts were measured at pH 3.5 for (A) 2,6‐DMP, (B) guaiacol, (C) syringaldazine and (D) at pH 3.0 for ABTS. The activity of r*Or*Lcc2‐D206N was also measured at pH 6.0 with all substrates. The activities are normalized with total protein concentrations. The measurements were carried out as triplicates. The error bars are 95% confidence interval.

The substrate specificities between the parental laccases and the variants were measured using phenolic substrates 2,6‐DMP, guaiacol and SGZ, and non‐phenolic ABTS (Fig. 2). The highest activities were detected with r*Or*Lcc2‐D206N for the phenolic substrates at pH 6.0. In addition, activity towards SGZ was detected only with r*Or*Lcc2‐D206N at pH 6.0. At the optimal pH conditions of the enzymes, r*Or*Lcc2‐D206N showed 6.8‐fold and 3.5‐fold higher activity towards 2,6‐DMP and guaiacol, respectively, than the parental r*Or*Lcc2 (Hildén *et al*., 2013). Also, the activity of r*Or*Lcc2‐D206N towards ABTS improved 10.6‐fold at pH 3.0 and 3.5‐fold at pH 6.0. The temperature tolerance of both laccase variants was observed to be substrate‐specific. Activity towards ABTS was more sensitive to increased temperatures than the more robust activity towards 2,6‐DMP (Fig. S2).

### Model compound oxidations by LMS

The LMS oxidation of two non‐phenolic lignin model compounds, the monomeric veratryl alcohol (**1**) and the dimeric β‐O‐4‐arylether adlerol (**3**) with benzylic α‐hydroxyl group, were studied to compare the catalytic efficiency and mediator preferences of the *O. rivulosa* laccases. Our results show that r*Or*Lcc2‐D206N variant retained as a high redox laccase at neutral pH, shown by its ability to oxidize N‐OH‐type mediators (Barreca *et al*., 2004) HBT, HPI, VIO and TEMPO (Table 2). r*Or*Lcc2‐D206N preferred TEMPO, HBT and SCN to mediate the oxidation of adlerol to the corresponding ketone, adlerone (**4**), whereas r*Or*Lcc1‐D208N showed preference for SCN and HBT. Oxidation of non‐phenolic substrates with phenolic mediators was faster by the *O. rivulosa* laccases than the commercial high‐redox potential NS51002 laccase (Novozymes A/S, Bagsvaerd, Denmark). In contrast, the oxidation with commecial N‐OH‐type mediators was less effective by the *O. rivulosa* laccases.

**Table 2:** Oxidation of adlerol to adlerone and veratryl alcohol (VerOH) to veratraldehyde (Fig. 5) as conversion % with r*Or*Lcc1, r*Or*Lcc1‐D208N, r*Or*Lcc2‐L497V, r*Or*Lcc2‐D206N and Novozymes 51002 in the presence of different mediators at 24 h reaction time.

	r*Or*Lcc1^a^		r*Or*Lcc1‐D208N		r*Or*Lcc2‐L497V^a^		r*Or*Lcc2‐D206N		NS51002	
	pH 3.0		pH 4.5		pH 3.0		pH 4.5		pH 4.5	
Substrate	Adlerol	VerOH	Adlerol	VerOH	Adlerol	VerOH	Adlerol	VerOH	Adlerol	VerOH
Mediator										
TEMPO	13	60	4	99	0	66	70^b^, ^d^	100	50	93
HBT	33	33	12	24	20	20	11	34	92	100
VIO	36	17	30	39	32	10	9	37	81	83
HPI	3	1	4	12	0	1	n.d.	11	45	70
SCN	n.d.	n.d.	9	42	3^c^	22^c^	17^d^	60^d^	9	61
MeS	2	2	2	9	2	1	8^e^	52^e^	6	38

^a^ Conversion yields obtained from Kontro et al. (2020).

^b^ Instead of adlerone, a product mixture was detected at pH 6.0, which most likely contained primary alcohol oxidation product (*γ*‐oxidized adlerol) together with low amounts of retro‐aldol side‐chain cleavage product of adlerol (veratraldehyde). The consumption of starting material suggested up to 70% adlerol conversion during 24 h.

^c^ Reaction was performed at pH 4.0.

^d^ Reaction was performed at pH 6.0.

^e^ Reaction was performed at pH 7.0.

Laccase variants r*Or*Lcc1‐D208N and r*Or*Lcc2‐D206N showed overall higher oxidation capacity for veratryl alcohol (**1**) than the parental laccases (Table 2). The highest conversion of veratryl alcohol to veratraldehyde (**2**), 60–66% for the parental laccases and 99–100% for variants, was obtained using TEMPO as a mediator. The phenolic mediators, MeS and SCN, are generally more stable and effective at near neutral pH, and r*Or*Lcc2‐D206N reached 50‐60% conversion yields at pH 6.0. The ability of the laccase variants to utilize environmentally benign phenolic mediators enable their use in large‐scale applications, whereas toxic side‐products formed during oxidation with the N‐OH‐type mediators and ABTS hamper their industrial use (Cañas and Camarero, 2010).

The mutations in laccase T1 substrate‐binding site are well‐known to introduce beneficial improvements in enzyme activity; however, the accumulation of beneficial mutations can also result in destabilization of the protein scaffold (Mate and Alcalde, 2015; Pardo and Camarero, 2015). The LMS experiments showed that the r*Or*Lcc2‐D206N variant had high oxidation capacity in a wide pH range. The more neutral reaction conditions stabilize the phenolic mediators, and thereby improve the reaction efficiency and turnover (Nousiainen *et al*., 2012). This is in line with the conversion yields of adlerol and veratryl alcohol with MeS and SCN by *Or*Lcc2‐D206N (Table 2).

### Depolymerization of biorefinery lignin

*Obba rivulosa* laccase variants in combination with selected redox mediators (Table 2) were examined for depolymerization of hot ethanol‐soluble fraction of hardwood biorefinery lignin. The M_N_ of the starting lignin fractions varied from 1114 to 1703 Da, and their polydispersity was 1.7, which showed that the fractions contained mostly macromolecules from pentamers to octamers, indicating that 25–40% of the lignin phenylpropane units occurred as end‐groups.

Direct laccase‐catalysed oxidation of lignin guaiacyl end‐groups typically produces reactive radical intermediates that further condense through self‐coupling forming new C‐C or C‐O bonds. Especially the high pH reaction conditions have been shown to result in an end‐wise coupling process and increased molecular weight of polymers (Gasser *et al*., 2017). Based on changes in molecular weight distribution, both laccase variants showed more depolymerization effect on lignin than the parental laccases (Table 3). The LMS generates selective modifications on the benzylic positions of lignin macromolecules and was not expected to affect the molecular weight distributions compared with the non‐mediated reactions. The low‐molecular weight mediators that more easily access the laccase active centre compete with the direct oxidation of the macromolecules, restricting the polymerization effect (Crestini *et al*., 2003). The highest depolymerization, resulting in ∆M_N_ −25% and −21%, was detected with r*Or*Lcc1‐D208N by using SCN and HBT as mediators, respectively (Fig. S4, Table S3). Also, r*Or*Lcc2‐D206N showed the most efficient depolymerization with SCN (∆M_N_ −17%) (Fig. S4, Tables 3 and S3). The overall changes in polydispersities were low, from 1.8 to 2.0, indicating that there was no apparent radical condensation through 5‐5’‐ or 5‐O‐4’‐bonds even without the use of mediators. This suggests that even with the increased solubility of larger macromolecules at higher pH, their access to the catalytic site of the mutated enzymes may be more restricted than in the parental enzymes. This slows down the formation of phenoxy radicals, regardless of the higher catalytic efficiency of the enzymes towards phenolic substrates. Our results indicate that the *O. rivulosa* laccase variants with selected mediators are more suitable for depolymerization of biorefinery lignin than the parental laccases (Kontro *et al*., 2020).

**Table 3:** Enzymatic depolymerization of hardwood lignin by r*Or*Lcc1, r*Or*Lcc1‐D208N, r*Or*Lcc2‐L497V and r*Or*Lcc2‐D206N with selected mediators and 20% 1,4‐dioxane as co‐solvent for mediators detected by gel permeation chromatography (GPC).

	r*Or*Lcc1^a^	r*Or*Lcc1‐D208N	r*Or*Lcc2‐L497V^a^	r*Or*Lcc2‐D206N
Mediator	VIO, SCN, HBT	VIO, SCN, TEMPO, HBT	VIO, SCN, TEMPO, HBT	VIO, SCN, TEMPO
pH	3.0	4.5	3.5	6.0
Effect on M_N_ (%)				
Lcc	+5	−9	+13	−4
LMS	+7 to −8	−7 to −25	+6 to +22	−10 to −17

Lcc, enzyme without mediators; LMS, laccase mediator system.

^a^ Results are obtained from Kontro *et al*. (2020).

**Fig. 3:**
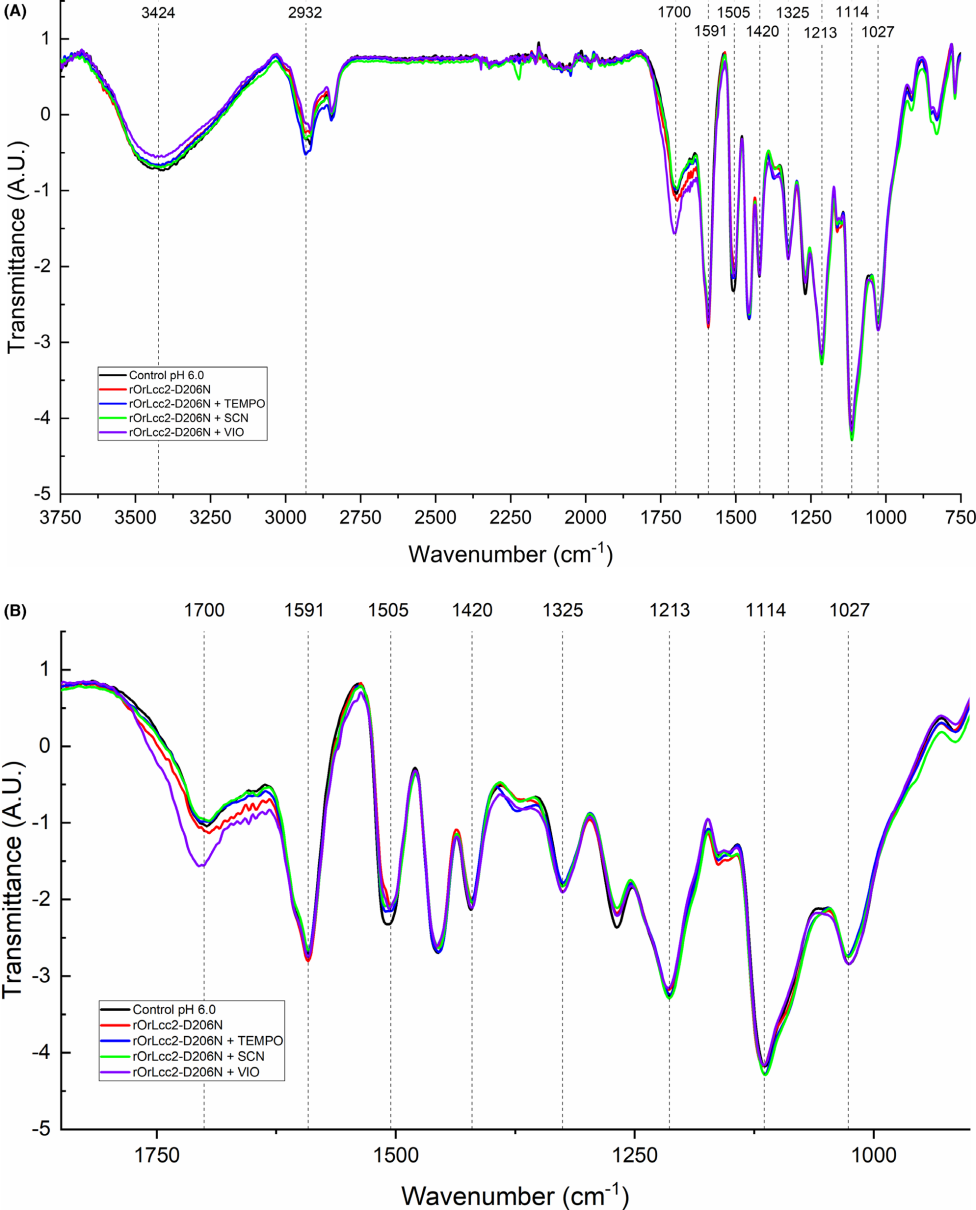
A. FTIR spectra of LMS‐oxidized lignin by r*Or*Lcc2‐D206N at pH 6.0; (B) the fingerprint region of IR spectra at 900–1800 cm^‐1^.

The structural changes in the LMS‐oxidized lignin samples were analysed by IR to verify the overall changes in the functional groups and molecular backbone. The highest lignin oxidation was detected with r*Or*Lcc2‐D206N and VIO, as showed by the signal increase at the conjugated carbonyl region (1650–1690 cm^−1^) (Fig. 3). The oxidation by LMS typically promotes an increase in carbonyl functionalities in the lignin benzylic position, together with a decrease in the hydroxyl content (Sammons *et al*., 2013). These results are in line with the FITR spectrum of the parental *Or*Lcc2 and VIO, where an increased amount of conjugated carbonyl signal was present (Kontro *et al*., 2020). In addition, a signal decrease was detected at the hydroxyl stretching area (3000–3600 cm^−1^) as well as at the aliphatic C–H stretching area (2934 cm^−1^). This supports the findings of increased lignin backbone oxidation at the side‐chain benzylic positions (Sammons *et al*., 2013). Compared with r*Or*Lcc2‐D206N, only low amounts of carbonyls were produced by r*Or*Lcc1‐D208N with HBT. Overall, the changes in the fingerprint region between 700 and 1500 cm^−1^ were minor, indicating that the molecular aromatic backbone of lignin was not significantly altered during LMS oxidation (Fig. S5).

Based on the FTIR results, the r*Or*Lcc2‐D206N LMS‐oxidized samples were analysed by NMR. The biorefinery lignin used in the oxidation experiments has been shown to contain syringyl (S) and guaiacyl (G) units at an S/G ratio of 2:1 and also γ‐esterified *p*‐hydroxybenzoate units (Kontro *et al*., 2020), thus resembling natural poplar lignin (Mansfield *et al*., 2012; Anderson *et al*., 2019). In addition, the major structural moieties of this lignin fraction are arylglycerol β‐arylether β‐O‐4 units, together with some phenylcoumaran β‐5 and resinol‐type β–β structures with a ratio of 6:1:1.3, respectively (Kontro *et al*., 2020).

**Fig. 4:**
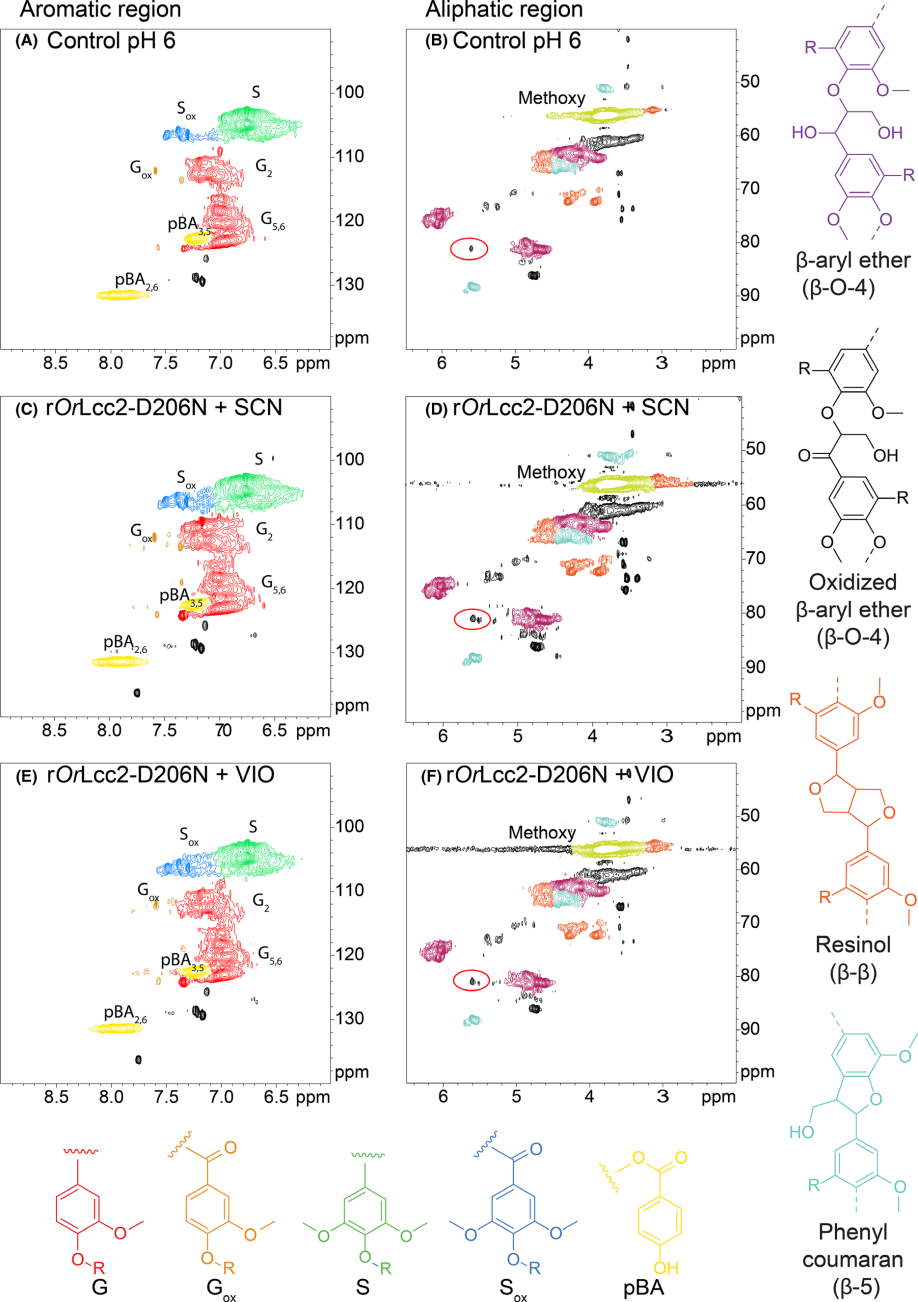
HSQC correlation spectra of r*Or*Lcc2‐D206N LMS‐oxidized lignin at aromatic region and aliphatic oxygenated side‐chain region. Syringyl nitrile (SCN) (C, D) and violuric acid (VIO) (E, F) were used as redox mediators. In the aliphatic oxygenated region, the shifted β‐proton signals in oxidized side‐chains are highlighted with red circles. Assignments of key structures are based on the literature (Kim and Ralph, 2010; Ralph and Landucci, 2010; Wei *et al*., 2013).

At the HSQC aromatic correlation region (δ_C_/δ_H_, 100–160/8.5–6.0 ppm), the oxidized benzylic positions could be most reliably detected by following the changes in the chemical shifts of S_2,6_ signals (δ_C_/δ_H_, 104.0/6.7 ppm) to oxidized S_2,6ox_ signals (δ_C_/δ_H_, 106.0/7.4 ppm). We detected that the LMS treatment by r*Or*Lcc2‐D206N with SCN and VIO clearly resulted in an increased ratio of these signal areas (S/S_ox_) compared with the control sample (Fig. 4C and E), which has been considered as a reliable indication of oxidative modifications of lignin (Sette *et al*., 2011). Previously, the S‐units of eucalyptus lignin have been reported to be oxidized by a low redox potential commercial fungal laccase (Novozym 51003) in the presence of a phenolic mediator MeS (Rico *et al*., 2014). In the control lignin sample, some oxidized correlation peaks were found as sharp signals indicating the presence of small‐molecular weight oxidized species before the LMS treatment, which can be formed during steam explosion pretreatment or originate from poplar wood extracts (Fig. 4). In addition, we detected some oxidation of G‐units by r*Or*Lcc2‐D206N with SCN and VIO (Fig. 4C and E). However, the correlations were scattered and ambiguous. Previously, with chemically treated lignins, the corresponding G_2,6_ signals at δ_C_/δ_H_, 110.8/7.0 and 120/6.9 ppm were found to be shifted to G_2ox_ at δ_C_/δ_H_, 110/7.5 ppm and G_6ox_ at δ_C_/δ_H_, 124/7.6 ppm (Kim and Ralph, 2010).

Selective benzylic oxidation resulting in the corresponding chemically more reactive α‐carbonyl structures was the most important modification in the biorefinery lignin fractions by the LMS treatment. The analysis of the aliphatic oxygenated lignin side‐chain region (δ_C_/δ_H_, 90–45/6.0–3.5 ppm) suggested that in case of arylglycerol β‐arylether β‐O‐4 bonds, the oxidation of benzylic alcohol groups to corresponding carbonyls by the LMS treatment shifted their adjacent β‐proton signals (4.3 ppm) to lower fields (Fig. 4D and F). The correlation peaks at 81/5.6 ppm and 82/5.5 ppm were attributed to these oxidized structures. Reference oxidations were performed using selective lignin benzylic oxidation by 2,3‐dichloro‐5,6‐dicyano‐1,4‐benzoquinone (DDQ) (Lancefield *et al*., 2015; Guo *et al*., 2018) for verification of the signals in similar NMR conditions. Visible changes in the amounts of phenylcoumaran β‐5 and resinol β‐β interconnecting structures could not be detected after LMS (Fig. 4).

Our LMS oxidation experiments with r*Or*Lcc2‐D206N clearly show promising modifications on lignin through benzylic oxidation as detected by IR and NMR. Importantly, no indication of increase in lignin molecular weight was found.

## Conclusions

We successfully changed the hydrophilic Asp208/Asp206 residues in the vicinity of the active site T1 of *O. rivulosa* laccases by replacing them with positively charged Asn. The laccase variant r*Or*Lcc2‐D206N showed significant structural modifications of biorefinery hardwood lignin in the presence of the mediators syringyl nitrile and violuric acid. The pH range of r*Or*Lcc2‐D206N shifted towards neutral by three pH units with the optimum at pH 6.0. This allowed the use of more neutral reaction conditions, which improved the solubilization of lignin. Selective benzylic oxidation through redox mediators and a decrease in molecular weight of lignin were observed, which is a promising result in terms of sustainable lignin valorization through further chemical and enzymatic treatment of lignin.

## Experimental procedures

### Microbial strains and site‐directed mutagenesis

*Pichia pastoris* strain X‐33 was purchased from Invitrogen (Gibco‐BRL, Gaithersburg, MD, USA). For heterologous expression, the codon‐optimized laccase variants in pPicZαA expression vector were purchased (GenScript, NJ, USA). Laccase variants *Or*Lcc1‐D208N and *Or*Lcc2‐D206N were designed based on the cDNAs‐encoding laccase isoenzymes *Or*Lcc1 and *Or*Lcc2 of *O*. *rivulosa* (previously *Physisporinus rivulosus*; GenBank JQ027726 and JQ027727). *Or*Lcc2‐D206N also had additional modification (L497V) at the C‐terminus, which has been shown to facilitate secretion when the possible ER‐retention signal SDEL was removed (Hildén *et al*., 2013).

### Heterologous expression of laccases in *P. pastoris*

The recombinant protein production in *P. pastoris* was carried out as fed‐batch cultivations in 5‐l Biostat B (B. Braun Biotech International Ag, Melsungen, Germany) equipped with pH and oxygen probes (Mettler Toledo, Columbus, OH, USA) mostly according to Invitrogen *Pichia* Fermentation Process Guidelines (Invitrogen, USA, 2002).

### Laccase activity and protein concentration assays

Laccase activities were determined spectrophotometrically by following the oxidation of 2,2′‐azino‐bis(3‐ethylbenzthiazoline‐6‐sulfonate) (ABTS), 2,6‐dimethoxyphenol (2,6‐DMP), syringaldazine (SGZ) and guaiacol at 420, 476, 525 and 465 nm, respectively (Leonowicz and Grzywnowicz, 1981; Paszczyhski *et al*., 1985; Bourbonnais and Paice, 1990; Slomczynski *et al*., 1995). The measurements were performed in 0.1 M citrate phosphate buffer at pH 3.0 for ABTS, and at pH 3.5 for 2,6‐DMP, SGZ and guaiacol. In addition, the activities of r*Or*Lcc2‐D206N towards different substrates were determined at pH 6.0. A Shimadzu UV‐1800 spectrophotometer was used in all the measurements. Laccase activity is expressed as μkat l^−1^ (10^‐6^ mol s^−1^ l^−1^) of the specific product formed upon oxidation of each substrate. The protein concentrations were measured by BCA Protein Assay Reagent (Pierce, Thermo Fisher Scientific, Rockford, IL, USA) using bovine serum albumin as standard according to the instructions of the manufacturer.

### Characterization of recombinant laccases

Steady‐state kinetic parameters (*K*_m_ and *k*_cat_) for purified r*Or*Lcc1‐D208N and r*Or*Lcc2‐D206N were determined by using 2,6‐DMP as a reducing substrate. Reactions were initiated by the addition of 0–31 mM 2,6‐DMP for r*Or*Lcc1‐D208N (pH 3.5) and 0–2 mM 2,6‐DMP (pH 6.0) for r*Or*Lcc2‐D206N. All measurements were performed in triplicates. The *K*_m_ and *V*_max_ values were estimated using MATLAB software via the Wilkinson nonlinear regression technique (Nelatury *et al*., 2014). The *k_cat_* values were calculated as *V*_max_/Lcc protein concentration.

The substrate specificities of r*Or*Lcc1‐D208N and r*Or*Lcc2‐D206N as well as the previously reported r*Or*Lcc1 and r*Or*Lcc2‐L497V (Hildén *et al*., 2013) were measured using 300 μM guaiacol at pH 3.5, 300 μM ABTS at pH 3.0, 33.3 μM SGZ at pH 6.0 and 300 μM 2,6‐DMP at pH 3.5 as reducing substrates. In addition, activities of r*Or*Lcc2‐D206N were measured with the different reducing substrates at pH 6.0, which was determined to be the optimal pH for this enzyme for oxidation of 2,6‐DMP. The activities between different enzymes were normalized with total protein concentrations for comparisons.

### LMS experiments with lignin model compounds

**Fig. 5:**
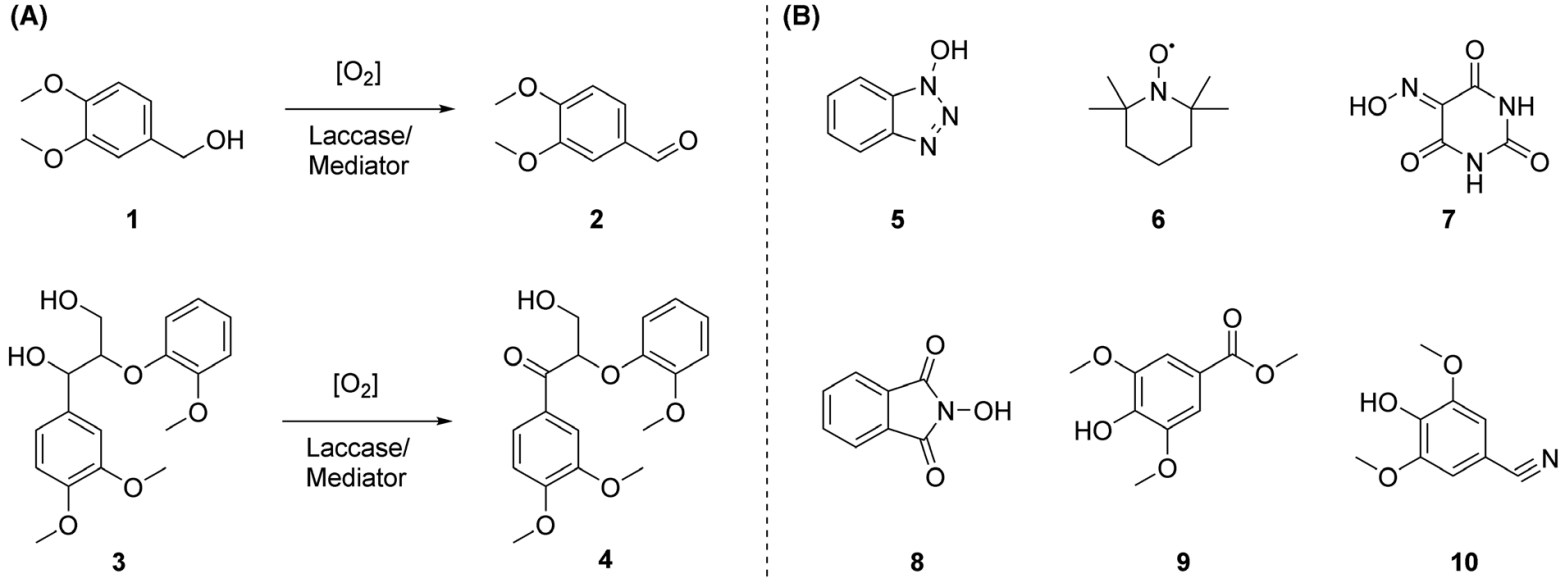
A. Oxidation of lignin model compounds veratryl alcohol (**1**) to veratraldehyde (**2**) and adlerol (**3**) to adlerone (**4**) by LMS; (B) chemical structures of mediators (**5‐10**) used in LMS experiments.

The mediator experiments were carried out using model compound reactions, as depicted in Fig. 5A: veratryl alcohol (**1**; VerOH) (Acros Organics) to veratraldehyde (**2**; VerAld) and dimeric 2‐(2‐methoxyphenoxy)‐3‐(3,4‐dimethoxyphenyl)propane‐1,3‐diol (**3**; adlerol) to 1‐(3,4‐dimethoxyphenyl)‐3‐hydroxy‐2‐(2‐methoxyphenyl)propane‐1‐one (**4**; adlerone) (Nakatsubo et al., 1975). The different mediators (Fig. 5B) tested were the commercial hydroxybenzotriazole (**5;** HBT, Sigma‐Aldrich), 2,2,6,6‐tetramethylpiperidin‐1‐yl)oxyl (**6;** TEMPO, Sigma‐Aldrich), violuric acid (**7;** VIO, Fluka) and N‐hydroxyphtalimide (**8;** HPI, Sigma‐Aldrich), and two synthesized mediators methyl syringate (**9;** MeS**)** and syringyl nitrile (**10;** SCN) described in Kontro *et al*. (2020). Each reaction mixture was shaken in an Eppendorf Thermomixer C (500 rpm) at 25°C in 100 mM citrate phosphate buffer with 20% 1,4‐dioxane (v/v) with r*Or*Lcc1 (at pH 3.0), r*Or*Lac2‐L497V (at pH 3.0), r*Or*Lcc1‐D208N (at pH 4.5) and r*Or*Lcc2‐D206N (at pH 4.5‐7.0). The commercial laccase preparation Novozymes 51002 (Novozymes) (at pH 4.5) was used as a reference. The starting substrate, enzyme and mediator concentrations were 6 mM, 10 nkat ml^‐1^ and 6 mM, respectively. Samples were taken at 0, 2, 4, 24 and 48 h, and the conversion yields were measured with HPLC Agilent 1200 (Santa Clara, CA, USA), as described earlier (Nousiainen *et al*., 2014).

### Oxidation of biorefinery lignin

Biorefinery hardwood lignin was received from Italian Bioproducts (IBP; Crescentino, Piedmont, Italy). The raw lignin contained 30 w‐% (dry) residual sugars, 55 w‐% (dry) Klason lignin, 2 w‐% (dry) ashes and 13 w‐% other impurities, with a moisture content of 67%. The experiments were carried out using the hot ethanol‐soluble lignin fraction that was purified, as described by Kontro *et al*. (2020). The depolymerization reaction of purified lignin (0.2 g) was performed with 35 nkat laccase and 0.15 mmol of mediator. The reactions were carried out in a Thermomixer C (600 rpm) at 25°C for 72 h in the presence of 20% dioxane at the same pH conditions as in the LMS experiments. For r*Or*Lcc1‐D208N, the reaction was performed at pH 4.5 and for r*Or*Lcc2‐D206N at pH 6.0. Controls without laccase were included. The oxidized lignins were isolated from reaction mixtures by concentrating and adjusting the pH to 1.5. The precipitated lignins were collected by centrifugation (3200 g, 10 min, 20°C). Lignin pellets were washed with water to remove salts and buffers, and extracted with ethyl acetate to remove mediators. The ethyl acetate fractions were combined and used to extract the aqueous supernatants and evaporated to dryness. All collected materials were dried under argon flow. For gel permeation chromatography (GPC) and nuclear magnetic resonance spectroscopy (NMR) analyses, the lignin samples were acetylated by incubating in 1:1 mixture of acetic anhydride and pyridine overnight at 50°C. The changes in molecular weight were assessed as shifts of the molecular weight (MW) distribution. The structural analyses were carried out by GPC, and IR and NMRspectroscopy.

### Gel permeation chromatography

Acetylated samples in tetrahydrofuran (THF) (1 mg ml^‐1^) were stirred for 20 h and filtered (0.2 µm GHP syringe filters; Waters, Milford, CT, USA). The GPC measurements were performed on an Agilent Infinity 1260 equipment using a set of Acquity APCTM XT 45 Å (1.7 µm, 4.6 × 150 mm) and XT 200 Å (2.5 µm, 4.6 × 150 mm) columns (Waters Corporation) in a series. The analyses were run in THF with detection by UV (280 nm) and refractive index (RI). Data processing was performed using Agilent GPC Add‐on. Set of eight polystyrene standards (Scientific Polymer Products and Fluka Analytical) was used for calibration, the relative molecular weights were calculated by software giving *M*_N_ (number‐average molecular weight), *M*_W_ (weight‐average molecular weight) and polydispersity index (PDI; *M*_W_/*M*_N_).

### Infrared spectral analysis

The IR spectra of the non‐acetylated samples were recordered with a Bruker Alpha FTIR spectrometer equipped with ATR module for sampling. Each measurement consisted of 16 scans with background correction. From each experiment, three samples were tested, and the results were averaged. The spectra were background‐corrected using asymmetric least squares fitting and standardized using Origin 2020 software (OriginLab Corporation, Northampton, MA, USA).

### Nuclear magnetic resonance spectroscopy

A Bruker Avance III 500 MHz FT‐NMR‐spectrometer with a Bruker 5mm BBO probe was used for ^1^H, ^13^C, heteronuclear single‐quantum coherence (HSQC, hsqcetgp) experiments. The measurements were performed with acetylated samples dissolved in acetone‐d_6_. The spectra were processed with Bruker TopSpin 4.0.5 software. Solvent signals (2.05/29.84 ppm) were used as reference. The peaks were assigned based on the literature (Gottlieb *et al*., 1997; Ralph and Landucci, 2010; Rahimi *et al*., 2013; Balakshin and Capanema, 2015).

## Funding Information

This research was supported by the European Commission Horizon 2020 project FALCON, grant no: 720918 (JW, CL, JKo, MK, PN, XW, JS and KH), Marie Curie ITN network SuBiCat FP7, grant no: 607044 (IB, PK and KH) and the Academy of Finland grants no: 297847 (JKo and JKu) and no: 308284 (MRM).

## Conflict of interest

None declared.

## Supporting information

**Fig. S1**. Purification steps of (A) r*Or*Lcc1‐D208N and (B) r*Or*Lcc2‐D206N analyzed by SDS‐PAGE. **(**A) Lane 1. PageRuler Plus Prestained 10–250 kDa protein ladder, Lane 2. Culture filtrate of r*Or*Lcc1‐D208N, Lane 3. Concentrated culture filtrate, Lane 4. Desalted and concentrated r*Or*Lcc1‐D208N after HiTrap purification, Lane 5. Desalted and concentrated r*Or*Lcc1‐D208N after MonoQ purification, Lane 6. BSA (0.02 mg ml^‐1^). (B) Lane 1. PageRuler Plus Prestained 10–250 kDa protein ladder, Lane 2. Culture filtrate of r*Or*Lcc2‐D206N, Lane 3. Concentrated culture filtrate, Lane 4. Desalted and concentrated r*Or*Lcc2‐D206N after HiTrap purification, Lane 5. Desalted and concentrated r*Or*Lcc1‐D208N after MonoQ purification, Lane 6. BSA (0.02 mg ml^‐1^).
**Fig. S2**. Thermotolerance of laccase variants. Thermotolerance of r*Or*Lcc1‐D208N using (A) ABTS and (B) 2,6‐DMP as substrates and thermotolerance of r*Or*Lcc2‐D206N using (C) ABTS and (D) 2,6‐DMP as substrates. The experimented temperatures were 40°C (*purple line)*, 50°C (*blue circle*), 60°C (*black diamond*), 70°C (*green triangle*), and 80°C (*red square*).
**Fig. S3**. (A) 2,6‐DMP at the active site T1 of *Or*Lcc2‐D206N. (B) 2,6‐DMP at the active site T1 of *Or*Lcc1‐D208N. The simulated models are presented as superimpositions with the corresponding original copper atoms containing homology model.
**Fig. S4**. The GPC chromatograms of enzymatic depolymerization of hardwood lignin by r*Or*Lcc1‐D208N and r*Or*Lcc2‐D206N with selected mediators and 20% 1,4‐dioxane as co‐solvent.
**Fig. S5**. The FTIR spectra of LMS‐oxidized lignin by r*Or*Lcc1‐D208N with different mediators at pH 4.5.
**Fig. S6**. HSQC‐spectra of (A) catalytic DDQ/tBuONO/O_2_ (B) stoichiometric DDQ oxidized lignin.
**Table S1**. Purification of laccase variants. Activities were measured using ABTS as substrate.
**Table S2**. Movement of 2,6‐DMP substrate from the active site determined *in silico*. *Or*Lcc1 and *Or*Lcc1‐D208N are calculated at pH 3.5. *Or*Lcc2 and *Or*Lcc2‐D206N are calculated at pH 3.5 and 6.0. The error range is given as 95% confidence interval.
**Table S3**. Effect on molecular weight distribution expressed as the difference ΔM_N_ or ΔM_W_ between non‐treated lignin fraction and laccase‐ or LMS‐treated samples.

**Appenidix S1**. Models and input parameters for MD simulations.

**Fig. S7**. OrLcc1 homology model of 3kw7 prepared to pH 3.5.

**Fig. S8**. OrLcc1D208N homology model of 3kw7 prepared to pH 3.5.

**Fig. S9**. OrLcc2 homology model of 2hrg prepared to pH 3.5.

**Fig. S10**. OrLcc2 homology model of 2hrg prepared to pH 6.

**Fig. S11**. OrLcc1hrgD206N homology model of 2hrg prepared to pH 3.5.

**Fig. S12**. OrLcc1hrgD206N homology model of 2hrg prepared to pH 6.
