# Supplementary material for: Depolymerization of biorefinery lignin by improved laccases of the white‐rot fungus Obba rivulosa
Source: Microb Biotechnol. 2021 Jul 26;14(5):2140–51. doi: 10.1111/1751-7915.13896 (PMC8449659; doi:10.1111/1751-7915.13896)
Supplement: Supplementary file 1 — Fig. S1. Purification steps of (A) rOrLcc1‐D208N and (B) rOrLcc2‐D206N analyzed by SDS‐PAGE. (A) Lane 1. PageRuler Plus Prestained 10–250 kDa protein ladder, Lane 2. Culture filtrate of rOrLcc1‐D208N, Lane 3. Concentrated culture filtrate, Lane 4. Desalted and concentrated rOrLcc1‐D208N after HiTrap purification, Lane 5. Desalted and concentrated rOrLcc1‐D208N after MonoQ purification, Lane 6. BSA (0.02 mg ml‐1). (B) Lane 1. PageRuler Plus Prestained 10–250 kDa protein ladder, Lane 2. Culture filtrate of rOrLcc2‐D206N, Lane 3. Concentrated culture filtrate, Lane 4. Desalted and concentrated rOrLcc2‐D206N after HiTrap purification, Lane 5. Desalted and concentrated rOrLcc1‐D208N after MonoQ purification, Lane 6. BSA (0.02 mg ml‐1). Fig. S2. Thermotolerance of laccase variants. Thermotolerance of rOrLcc1‐D208N using (A) ABTS and (B) 2,6‐DMP as substrates and thermotolerance of rOrLcc2‐D206N using (C) ABTS and (D) 2,6‐DMP as substrates. The experimented temperatures were 40°C (purple line), 50°C (blue circle), 60°C (black diamond), 70°C (green triangle), and 80°C (red square). Fig. S3. (A) 2,6‐DMP at the active site T1 of OrLcc2‐D206N. (B) 2,6‐DMP at the active site T1 of OrLcc1‐D208N. The simulated models are presented as superimpositions with the corresponding original copper atoms containing homology model. Fig. S4. The GPC chromatograms of enzymatic depolymerization of hardwood lignin by rOrLcc1‐D208N and rOrLcc2‐D206N with selected mediators and 20% 1,4‐dioxane as co‐solvent. Fig. S5. The FTIR spectra of LMS‐oxidized lignin by rOrLcc1‐D208N with different mediators at pH 4.5. Fig. S6. HSQC‐spectra of (A) catalytic DDQ/tBuONO/O2 (B) stoichiometric DDQ oxidized lignin. Table S1. Purification of laccase variants. Activities were measured using ABTS as substrate. Table S2. Movement of 2,6‐DMP substrate from the active site determined in silico. OrLcc1 and OrLcc1‐D208N are calculated at pH 3.5. OrLcc2 and OrLcc2‐D206N are calculated at pH 3.5 and 6.0. The error range i [file MBT2-14-2140-s005.docx]

Supporting Information

Depolymerization of biorefinery lignin by improved laccases of the white rot fungus *Obba rivulosa*

Janne Wallenius^1,⁑^, Jussi Kontro^2,⁑^, Christina Lyra^1^, Jaana Kuuskeri^1^, Xing Wan^1^, Mika A. Kähkönen^1^, Irshad Baig^3,§^, Paul C.J. Kamer^3,^ , Jussi Sipilä^2^, Miia R. Mäkelä^1^, Paula Nousiainen^2^, Kristiina Hildén^1,^*

^1^Fungal Genetics and Biotechnology, Department of Microbiology, University of Helsinki, Biocenter 1, P.O. Box 56, Viikinkaari 9, FI-00014 University of Helsinki, Finland

^2^ Department of Chemistry, University of Helsinki, P.O. Box 55, A. I. Virtasen Aukio 1, FI-00014 University of Helsinki, Finland

^3^ EaStCHEM, School of Chemistry, University of St Andrews, Fife, Scotland, UK

*Corresponding author: Kristiina Hildén, e-mail: [kristiina.s.hilden@helsinki.fi](mailto:kristiina.s.hilden@helsinki.fi)

**Protein purification**

The chromatographic purification of the laccase variants resulted in 8.6% and 1.5% yield for r*Or*Lcc1-D208N and r*Or*Lcc2-D206N, respectively (Table S1). The purification fold of r*Or*Lcc1-D208N was 44.4 and that of r*Or*Lcc2-D206N 5.8. Protein fractions from the different steps of the purification are shown in Fig. S1.


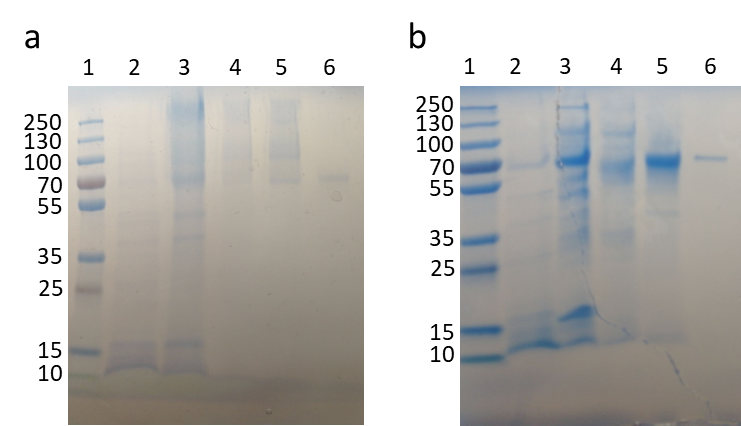


**Fig. S1** Purification steps of (A) r*Or*Lcc1-D208N and (B) r*Or*Lcc2-D206N analyzed by SDS-PAGE. **(**A) Lane 1. PageRuler Plus Prestained 10-250 kDa protein ladder, Lane 2. Culture filtrate of r*Or*Lcc1-D208N, Lane 3. Concentrated culture filtrate, Lane 4. Desalted and concentrated r*Or*Lcc1-D208N after HiTrap purification, Lane 5. Desalted and concentrated r*Or*Lcc1-D208N after MonoQ purification, Lane 6. BSA (0.02 mg/mL). (B) Lane 1. PageRuler Plus Prestained 10-250 kDa protein ladder, Lane 2. Culture filtrate of r*Or*Lcc2-D206N, Lane 3. Concentrated culture filtrate, Lane 4. Desalted and concentrated r*Or*Lcc2-D206N after HiTrap purification, Lane 5. Desalted and concentrated r*Or*Lcc1-D208N after MonoQ purification, Lane 6. BSA (0.02 mg/mL).

| **Table S1.** Purification of laccase variants. Activities were measured using ABTS as substrate. | | | | | |
| --- | --- | --- | --- | --- | --- |
| **r*Or*Lcc1-D208N** | **Protein (mg)** | **Total activity (nkat)** | **Specific activity (nkat/mg)** | **Yield (%)** | **Purification (fold)** |
| **Culture broth** | 2015.7 | 17347.3 | 8.6 | 100.0 | 1.0 |
| **HiTrap** | 22.0 | 4095.6 | 186.0 | 23.6 | 21.6 |
| **MonoQ** | 3.9 | 1488.5 | 382.2 | 8.6 | 44.4 |
| **r*Or*Lcc2-D206N** | **Protein (mg)** | **Total activity (nkat)** | **Specific activity (nkat/mg)** | **Yield (%)** | **Purification (fold)** |
| **Culture broth** | 1537.6 | 21953.3 | 14.3 | 100.0 | 1.0 |
| **HiTrap** | 31.2 | 1018.9 | 32.6 | 4.6 | 2.3 |
| **MonoQ** | 4.0 | 327.6 | 82.4 | 1.5 | 5.8 |

**Thermotolerance of laccase variants**

The temperature tolerance was observed to be substrate specific. Activity towards ABTS was more sensitive to increased temperatures (Fig. S2 A and C) compared to the more robust activity towards 2,6-DMP (Figure S2 B and D). The ABTS activity of r*Or*Lcc2-D208N was stable below 50°C while decrease in ABTS activity for the parental r*Or*Lcc2 has been observed already at 40°C (Hildén *et al*., 2013). Also r*Or*Lcc1-D208N variant exhibited higher temperature tolerance compared to the parental laccase (Hildén *et al*., 2013) obtained stable activities for 2,6-DMP at 50°C for both r*Or*Lcc1 and r*Or*Lcc2, while both the mutants exhibited stable activities at 60°C. Considering the temperature profile difference between the two substrates, the results suggest that more intact enzyme is required for ABTS activity while 2,6-DMP oxidation is more robust regarding the enzyme conformation.
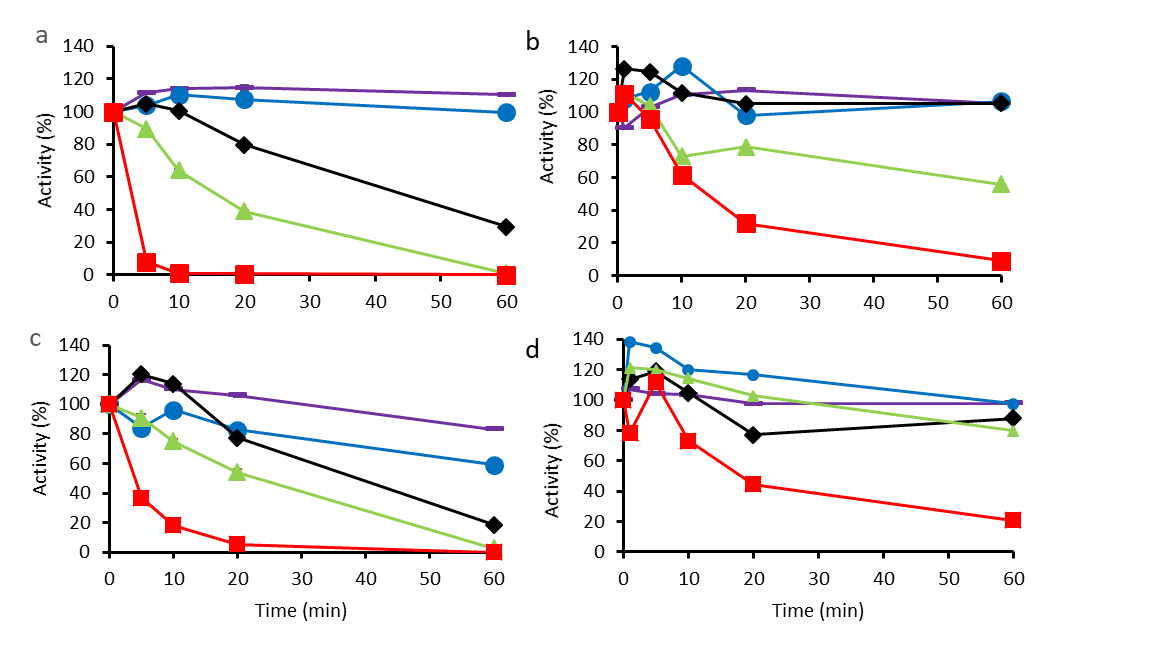
**Fig. S2** Thermotolerance of laccase variants. Thermotolerance of r*Or*Lcc1-D208N using (a) ABTS and (b) 2,6-DMP as substrates and thermotolerance of r*Or*Lcc2-D206N using (c) ABTS and (d) 2,6-DMP as substrates. The experimented temperatures were 40°C (*purple line)*, 50°C (*blue circle*), 60°C (*black diamond*), 70°C (*green triangle*), and 80°C (*red square*).

***In silico* analyses of *Or*Lcc1-D208N and *Or*Lcc2-D206N**

We applied molecular docking followed by MD simulations and subsequentially binding affinity analyses with MMGBSA over MD trajectories to investigate the interactions between the substrate and T1 active site of r*Or*Lcc1-D208N and r*Or*Lcc2-D206N. The laccase catalyzed oxidation of phenols is dependent on the difference of the redox potential between phenolic substrate and T1 center and correlates with the reaction rate (Xu, 1987). In addition, fungal laccases are typically dependent on acidic pH. With the elevated reaction pH, electron traffic from T1 center to T2/T3 center is inhibited by the increase of competing hydroxyl ions, which decrease laccase activity.

**Table S2.** Movement of 2,6-DMP substrate from the active site determined *in silico*. *Or*Lcc1 and *Or*Lcc1-D208N are calculated at pH 3.5. *Or*Lcc2 and *Or*Lcc2-D206N are calculated at pH 3.5 and 6.0. The error range is given as 95% confidence interval.

|  | **Enzyme** | | | |
| --- | --- | --- | --- | --- |
|  | ***Or*Lcc1** | ***Or*Lcc1-D208N** | ***Or*Lcc2** | ***Or*Lcc2-D206N** |
| **Substrate RMSF (Å)** | 11.4 | 7.9 | 6.1 / 57.2 | 13.8 / 15.4 |
| **MMGBSA binding free energies (kcal mol^-1^)** | -15.5 ± 3.4 | -17.1 ± 4.1 | -22.1 ± 4.0 / -8.0 ± 4.6 | -25.2 ± 4.1 / -15.3 ± 2.9 |

The T1 site of all laccases is well accessible for small phenolic 2,6-DMP molecules. The averaged binding affinities over the measured trajectories were higher for the laccase mutants compared to the corresponding unmodified enzyme. The asparagine mutation of *Or*Lcc2-D206N seemed to stabilize 2,6-DMP at the T1 active site (Fig. S3) and higher binding affinities for *Or*Lcc2-D206N were also detected experimentally at pH 3.5 and pH 6.0 compared to *Or*Lcc2. The binding affinity for *Or*Lcc2-D206N was higher at pH 3.5 while the measured laccase activity was higher at pH 6.0. Previously, it has been shown that low *K_m_* values correlate with the high binding affinities in laccases, and more specifically to the lifetime of active substrate pose within the enzyme (Mehra *et al*., 2018). The oxidation potential of 2,6-DMP, decreases significantly with increasing pH resulting in high electron transfer rate to the T1 copper at pH 6.0. This explains the high experimental activity even with the less stable active binding pose of 2,6-DMP. In contrast to our results with respect to enhanced 2,6-DMP activity by *Or*Lcc2-D206N (Madzak *et al*., 2006), obtained impaired 2,6-DMP activity with *Trametes versicolor* D206N laccase variant. MD simulations has suggested that the decrease of activity could be due to premature movement of 2,6-DMP from the T1 active site (Mehra *et al*., 2018). In laccase variant *Or*Lcc1-D208N, the MD simulation indicated that 2,6-DMP remained apart from the 208 residue at the T1 active site. All our MD simulations showed relatively rapid release of 2,6-DMP (within 20 ns), compared to the simulations with laccases by Mehra *et al*. (2018). The movement of the ligand from the active site as root-mean-square fluctuation (RMSF) of the substrate to the first frame of the simulation could not be directly related to the *K_m_* values.


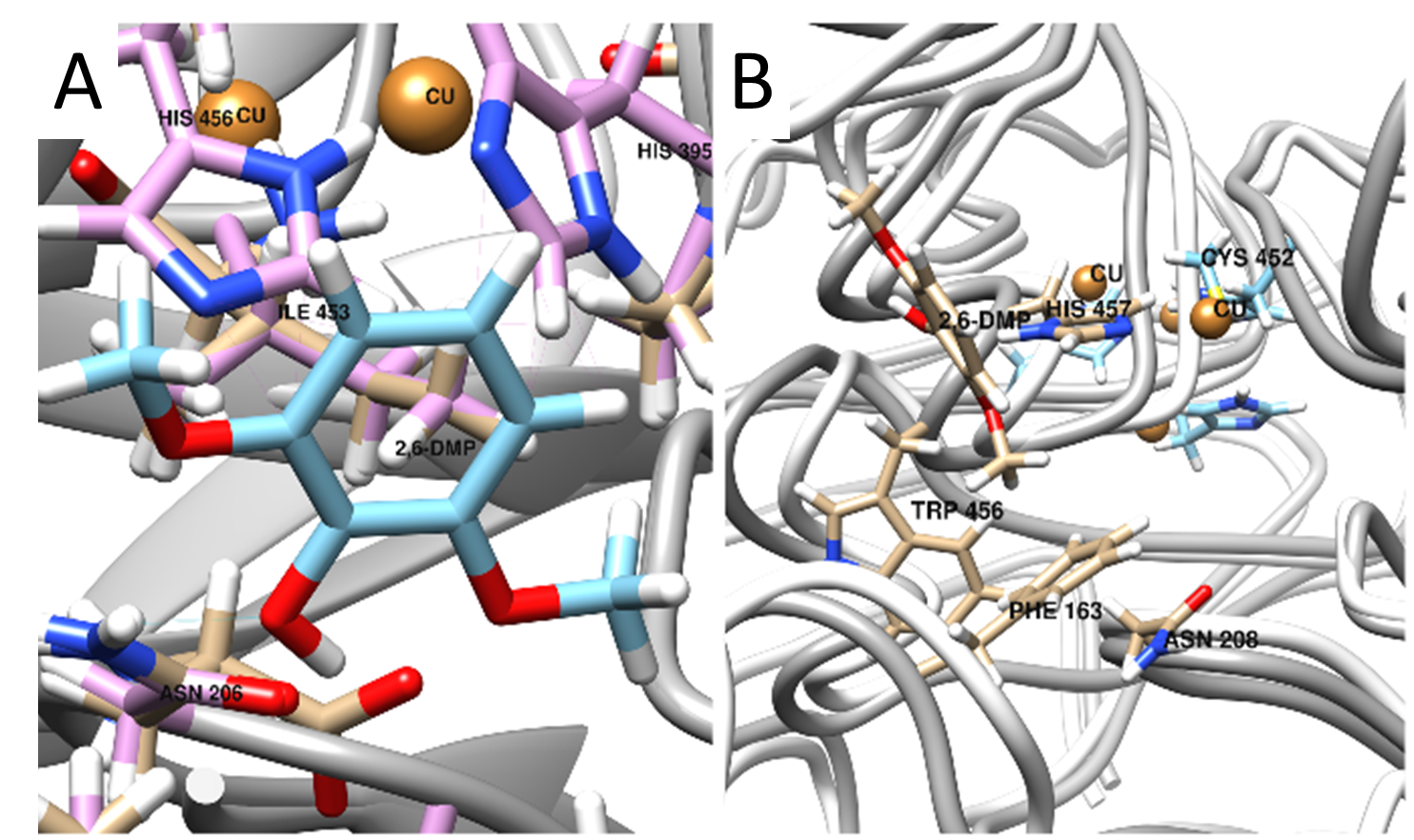


**Figure S3.** (A) 2,6-DMP at the active site T1 of *Or*Lcc2-D206N. (B) 2,6-DMP at the active site T1 of *Or*Lcc1-D208N. The simulated models are presented as superimpositions with the corresponding original copper atoms containing homology model.

**GPC chromatograms**

The de- and repolymerization of lignin was evaluated by GPC and the relative molecular weights were calculated by the software giving M_N_ (number-average molecular weight), M_W_ (weight-average molecular weight) and polydispersity index (PDI; M_W_/M_N_). Fig. S4 shows the GPC chromatograms of lignin oxidized by r*Or*Lcc1-D208N and r*Or*Lcc2-D206N with different mediators at pH 4.5 and 6.0, respectively. The corresponding numeral values are presented in Table S3.
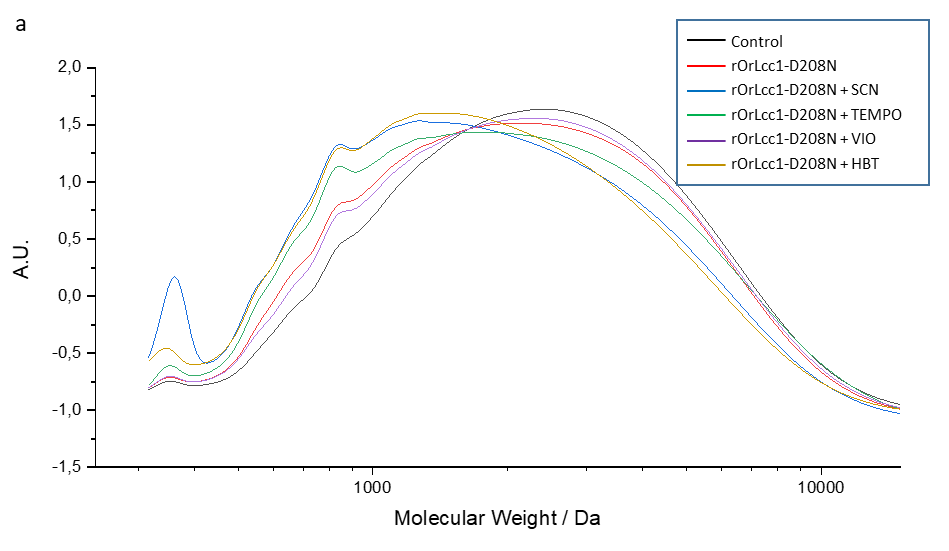


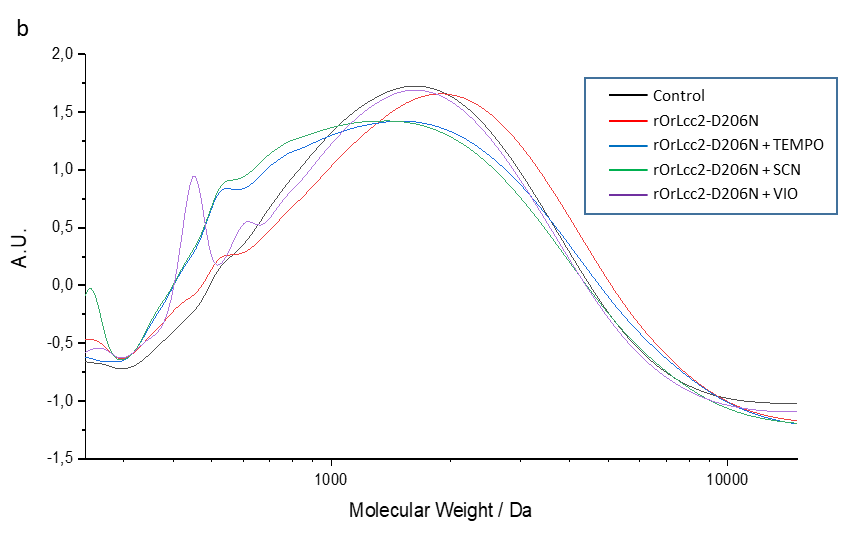


**Fig. S4** The GPC chromatograms of enzymatic depolymerization of hardwood lignin by r*Or*Lcc1-D208N and r*Or*Lcc2-D206N with selected mediators and 20% 1,4-dioxane as co-solvent.

**Table S3.** Effect on molecular weight distribution expressed as the difference ΔM_N_ or ΔM_W_ between non-treated lignin fraction and laccase- or LMS-treated samples.

|  | M_N_ | M_W_ | PDI | ΔM_N_ | ΔM_W_ | ΔM_N_-% | ΔM_W_-% |
| --- | --- | --- | --- | --- | --- | --- | --- |
| Control | 1703 | 2950 | 1.73 | 0 | 0 | 0 | 0 |
| r*Or*Lcc1-D208N | 1555 | 2781 | 1.79 | -148 | -169 | -9 | -6 |
| r*Or*Lcc1-D208N + SCN | 1280 | 2450 | 1.91 | -423 | -500 | -25 | -17 |
| r*Or*Lcc1-D208N + TEMPO | 1453 | 2818 | 1.94 | -250 | -132 | -15 | -4 |
| r*Or*Lcc1-D208N + VIO | 1589 | 2840 | 1.79 | -114 | -110 | -7 | -4 |
| r*Or*Lcc1-D208N + HBT | 1337 | 2403 | 1.80 | -366 | -547 | -21 | -19 |
|  |  |  |  |  |  |  |  |
| Control | 1114 | 1898 | 1.70 | 0 | 0 | 0 | 0 |
| r*Or*Lcc2-D206N | 1075 | 2142 | 1.99 | -39 | 244 | -4 | 13 |
| rOrLcc2-D206N + SCN | 928 | 1847 | 1.99 | -186 | -51 | -17 | -3 |
| r*Or*Lcc2-D206N + TEMPO | 998 | 2026 | 2.03 | -116 | 128 | -10 | 7 |
| r*Or*Lcc2-D206N + VIO | 1008 | 1829 | 1.81 | -106 | -69 | -10 | -4 |

**The FTIR spectra of LMS-oxidized lignin by r*Or*Lcc1-D208N**

The structural changes of the LMS-oxidized lignin samples were analyzed by IR to verify the overall changes in the functional groups and molecular backbone (Fig. S5). r*Or*Lcc1-D208N with HBT produced only low amounts of carbonyls compared to r*Or*Lcc2-D206N with different mediators.


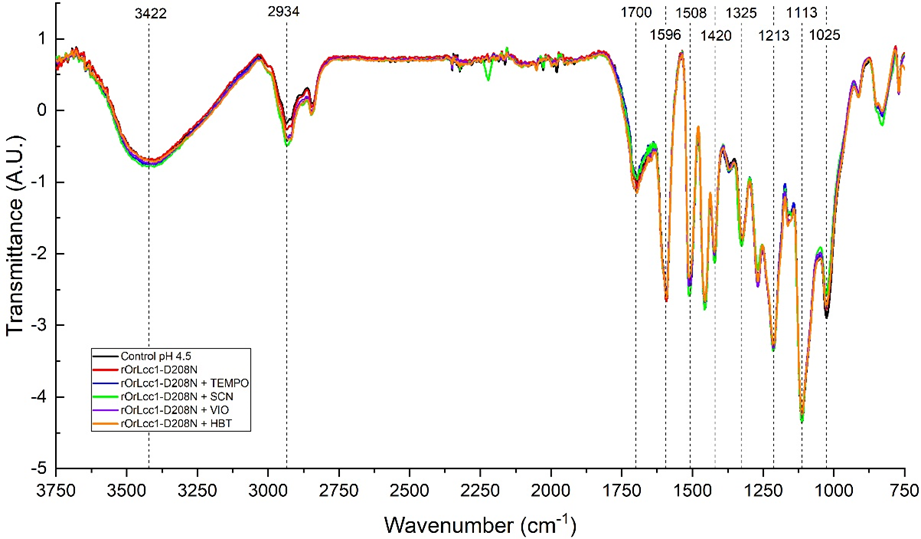


**Fig. S5** The FTIR spectra of LMS-oxidized lignin by r*Or*Lcc1-D208N with different mediators at pH 4.5.

**HSQC-spectra of DDQ oxidized lignin**

The reference oxidations for NMR analysis were performed by selective benzylic oxidation using 2,3-dichloro-5,6- dicyano-1,4-benzoquinone (DDQ; Aldrich) as oxidant (Lancefield *et al*., 2015; Guo *et al*., 2018). The DDQ has been reported as effective and selective two electron oxidant for lignin, but it suffers from high toxicity that hinders the larger scale applications.

The catalytic DDQ-oxidation of lignin was performed by using the catalytic DDQ/tBuONO/O_2_ and stoichiometric DDQ oxidization. In the aliphatic oxygenated region of both spectra, the shifted β-proton signals in oxidized side chains appear at δ_C_/δ_H_ 80.8/5.7 ppm and 81.3/5.6 ppm (Fig. S6). According to HSQCTOCSY the β-signals correlate to γ-region at 64/4.5 ppm. The aromatic oxygenated S2/6ox correlations show as a wide signal at δ_C_/δ_H_ 106.6/7.4 ppm and the corresponding correlations of G2ox at 112.9/7.7 ppm and G6ox at 123.2/7.7 ppm. In catalytic DDQ/ t-BuONO/O_2_ system, the oxidation efficiency was lower (Fig. S6 A) than in stoichiometric DDQ system (Fig. S6 B), and all original sub-structures can be found in the spectra together with oxidized counterparts. In the samples oxidized by stoichiometric amount of DDQ, all the signals of β-5 and β-β and majority of β-O-4 signals have disappeared compared to original lignin that indicates the efficiency of this oxidant.


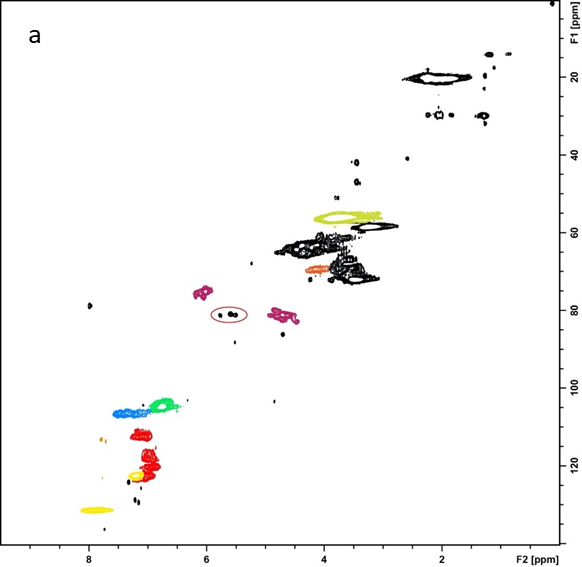


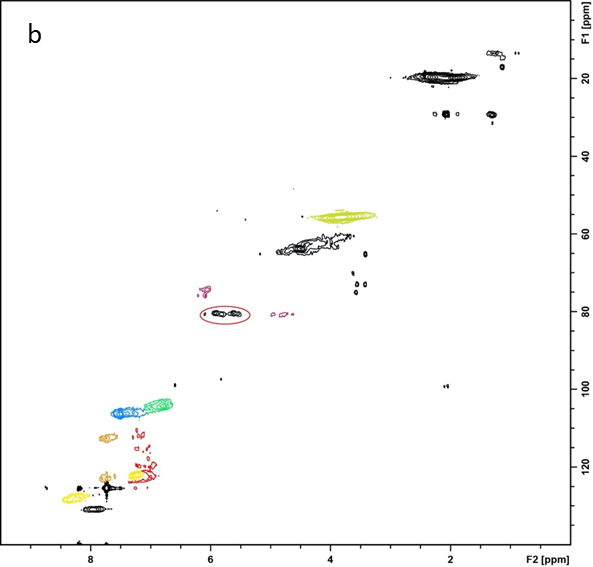


**Fig S6**. HSQC-spectra of A) catalytic DDQ/tBuONO/O_2_ B) stoichiometric DDQ oxidized lignin.

**Experimental procedures**

**Heterologous expression of laccases in *P. pastoris***

The recombinant protein production in *P. pastoris* was carried out as fed-batch cultivations in 5 L Biostat B (B. Braun Biotech International Ag, Germany) equipped with pH and oxygen probes (Mettler Toledo, Columbus, Ohio, U.S.) according to Invitrogen *Pichia* Fermentation Process Guidelines (Invitrogen, 2002) with the slight modifications. Briefly, recombinant laccases expressing *P. pastoris* (Mut^+^) were cultivated in 5 × 50 mL YPG (10 g/L yeast extract, 20 g/L peptone, 30 g/L glycerol) at 28°C with 180 rpm agitation to reach OD_600_ value between 1 to 10. Cells were harvested by centrifugation at 12,000 rcf for 10 min and resuspended to equal volume of basal salts medium (26.7 mL/L 85% H_3_PO_4_, 1.176 g/L CaSO_4_ × 2 H_2_O, 18.2 g/L K_2_SO_4_, 14.9 g/L MgSO_4_ × 7 H_2_O, 4.13 g/L KOH, and 40 g/L glycerol).

The initial growth medium for batch phase cultivation consisted of 2.5 L basal salts medium containing 4.0 w-% glycerol and 0.435 v-% PTM1 trace salts (6 g/L CuSO_4_ × 5 H_2_O, 0.08 g/L NaI, 3 g/L MnSO_4_ × H_2_O, 0.2 g/L Na_2_MoO_4_ × 2 H_2_O, 0.02 g/L H_3_BO_3_, 0.916 g/L CoCl_2_ × 6 H_2_O, 20 g/L ZnCl_2_, 65 g/L FeSO_4_ × 7 H_2_O, 0.2 g/L biotin, and 5 mL/L H_2_SO_4_). The pH was controlled to 4.95 with a dead band of 0.1 by addition of 15 v-% ammonia. The dissolved oxygen concentration was controlled to a setpoint of 25% pO_2_ by a PID controller setting the stirring speed of the bioreactor. The air flow was kept in between 0.75–1.00 vvm and temperature at 30°C. To control the foaming, antifoam agent Struktol J 647, was added as required. After the depletion of the glycerol in the batch phase, glycerol fed-batch phase was started introducing 50 w-% glycerol containing 1.2 v-% PTM1 trace salts at a rate of 20.5 mL/h. The glycerol fed-batch was continued until cell wet weight between 115 to 125 g/L (after 7–9 h) was achieved. Subsequently, the feed was stopped and dissolved oxygen was allowed to increase to confirm the depletion of carbon source. To induce the recombinant laccase expression, methanol containing 1.2 v-% PTM1 trace salts was introduced at the rate of 1 to 7 mL/h. The methanol flow was adjusted based on the requirement that the oxygen consumption was limited by methanol availability to ensure aerobic conditions and to prevent the toxic methanol accumulation.

**Purification of recombinant laccases**

The cultivation media of *P. pastoris* were centrifuged at 3000 rcf for 10 min at 4°C to remove the cells. The supernatant was filtered through Whatman filter paper under vacuum and subsequently concentrated using 100 kDa cutoff membrane in tangential filtration at 4°C followed by Amicon stirred cells (Merck, New Jersey, USA) using 10 kDa cutoff membrane under nitrogen pressure. The extracellular protein concentrates were stored at +4°C. The concentrates of r*Or*Lcc1-D208N and r*Or*Lcc2-D206N were purified by HPLC (the ÄKTA Explorer, GE Healthcare, Uppsala, Sweden) in two ion exchange chromatography columns (GE Healthcare Life Sciences, Uppsala, Sweden). First step was performed by HiTrap Q XL 1 mL column with 0-100% gradient of elution buffer (Buffer A: 10 mM triethanolamine (TEA); Buffer B: 10 mM TEA + 1 M NaCl, pH 7.0) and the second step by using MonoQ 5/50 GL with 0-40% gradient of elution buffer (Buffer A: 20 mM Tris-HCl; Buffer B: 20 mM Tris-HCl + 1 M NaCl, pH 8.0). The fractions showing laccase activity were collected, pooled and desalted with centrifugal concentrators (Spin-X UF 20 mL Centrifugal Concentrator, 10,000 MWCO Membrane, Corning Inc., Corning, New York, USA) between the chromatographic steps. The purity of the enzymes was analysed by SDS-PAGE (12% MiniProtean TGX gels; Bio-Rad, Hercules, USA) by applying five micrograms of purified proteins on the gel. The proteins were visualized by PageBlue (Fermentas) gel staining reagent. PageRuler Plus Prestained 10-250 kDa protein ladder (Thermo Scientific, Massachusetts, USA) was used for the determination of the molecular mass.

**Thermotolerance of laccase variants**

The thermal tolerance of the laccases was determined by measuring the residual enzyme activity with ABTS and 2,6-DMP at temperatures from 40 to 80°C at the pH 3.0 and 3.5, respectively. Activities were measured after 0 to 60 min of incubation at given temperature. The activity at 40°C without incubation was set as 100% activity. The pH range of the enzymes was determined for ABTS and 2,6-DMP by measuring the activities as described above at pH values from 2.5 to 7.0. The measurements were carried out as triplicates and averages are plotted.

**Modeling, simulations and binding affinities**

The homology models for *Or*Lcc1 and *Or*Lcc2 enzymes and the variants *Or*Lcc1-D208N and *Or*Lcc2-D206N were constructed with SWISS-MODEL using crystal structures of laccases PDB ID: 3KW7 (*Trametes* sp. AH28-2) and 2HRG (*Trametes trogii*) from the Protein Data Bank for *Or*Lcc1/*Or*Lcc1-D208N and *Or*Lcc2/*Or*Lcc2-D206N, respectively (Waterhouse *et al*., 2018). The mature translated amino acid sequences, from which the predicted signal peptides were omitted, were used for alignment with their homologues as presented previously (Hildén *et al*., 2013). The models were prepared to correspond the pH 3.5 and 6.0 conditions with ProteinPrepare web application and also substrate structures were curated accordingly (Martínez-Rosell *et al*., 2017). The models were docked with the substrates using SwissDock constraining the docking area close to the assumed active site T1 (Grosdidier *et al*., 2011). Flexibility for side chains within 3 Å of any atom of the substrate in its reference binding mode was allowed for the docking of *Or*Lcc1/*Or*Lcc1-D208N.

Suitable docking poses at the active site were used as a starting point for molecular dynamics (MD) simulations. The substrate files and the models were further prepared and subsequently simulated with AmberTools (Case *et al*., 2018). The substrate files were prepared with ANTECHAMBER module of AmberTools. The protein-ligand complexes along with the Amber parameter and coordinate files for MD simulations were prepared from Cu atom excluded PDB files of the enzyme models with LEaP module applying ff99 force field and mbondi2 for atomic radii. The protein-ligand complexes were neutralized with additions of Na^+^ or Cl^-^ and solvated with TIP3P water model. The MD simulations for the solvated complexes were carried out stepwise, briefly: minimization, heating and density equilibration, constant pressure equilibration and production with sander or GPU-accelerated pmemd engines. The input files for MD simulations are given in Supporting information 2. CPPTRAJ was used in trajectory data processing (Roe and Cheatham, 2013). The binding affinities were assessed as average over trajectory using MMPBSA.py with a generalized born solvent model (igb = 2) and 0.1 M salt concentration (Miller *et al*., 2012). The visualization was carried out using Chimera software (Pettersen *et al*., 2004).

**DDQ-oxidation**

The catalytic DDQ-oxidation was performed according modified method of Lancefield *et al*. (2015). The ethanol soluble poplar lignin (0.5 g) was dissolved in 6 ml 2-methoxyethanol, and 60 µl *tert*-Butyl nitrite (t-BuONO; Aldrich) reagent and 0.05 g DDQ were added. The reaction mixture was heated at 80 °C for 18 h under oxygen atmosphere. The solution was cooled and 50 ml diethyl ether was added to precipitate lignin. The precipitate was filtered through PTFE filter and the solids were washed with diethyl ether. The solids were mixed with NaHCO_3_ solution, filtered and washed with water. The residue was dried, acetylated and the NMR spectra were measured in acetone-d_6_.

The ethanol soluble poplar lignin was oxidized with DDQ in 1,4-dioxane. One equivalent of reagent was used according to approximation of the lignin fractions phenylpropanol units. 0.2 g lignin was dissolved in hot 1,4-dioxane (12 ml) and 0.2 g DDQ was added. The mixture was stirred during 18 h reaction time at 60 °C after which the mixture was cooled and centrifuged to separate the reduced reagent. The solution was then poured to diethyl ether to precipitate lignin. The precipitate was washed with ether and the residue was dried, acetylated and the NMR spectra were measured in acetone-d_6_.

**References**

Case, D.A., Ben-Shalom, I.Y., Brozell, S.R., Cerutti, D.S., Cheatham, T.E.I. *et al*. (2018) AMBER 2018, University of California, San Francisco

Grosdidier, A., Zoete, V., and Michielin, O. (2011) SwissDock, a protein-small molecule docking web service based on EADock DSS. *Nucleic Acids Res* **39**: 270–277.

Guo, H., Miles-Barrett, D.M., Neal, A.R., Zhang, T., Li, C., and Westwood, N.J. (2018) Unravelling the enigma of lignin^OX^: can the oxidation of lignin be controlled? *Chem Sci* **9**: 702–711.

Hildén, K., Mäkelä, M. R., Lundell, T., Kuuskeri, J., Chernykh, A., Golovleva, L., Archer, D.A., and Hatakka, A. (2013) Heterologous expression and structural characterization of two low pH laccases from a biopulping white-rot fungus *Physisporinus rivulosus*. *Appl Microbiol Biotechnol* **97**: 1589–1599.

Lancefield, C. S., Ojo, O. S., Tran, F., and Westwood, N.J. (2015) Isolation of functionalized phenolic monomers through selective oxidation and C-O bond cleavage of the β-O-4 linkages in lignin. *Angew Chemie Int Ed* **54**: 258–262.

Madzak, C., Mimmi, M. C., Caminade, E., Brault, A., Baumberger, S., Briozzo, P., Mougin, C., and Jolivalt, C. (2006) Shifting the optimal pH of activity for a laccase from the fungus *Trametes versicolor* by structure-based mutagenesis. *Protein Eng Des Sel* **19**: 77–84.

Martínez-Rosell, G., Giorgino, T., and De Fabritiis, G. (2017) PlayMolecule ProteinPrepare: A web application for protein preparation for molecular dynamics simulations. *J Chem Inf Model* **57**: 1511–1516.

Mehra, R., Meyer, A.S., and Kepp, K.P. (2018) Molecular dynamics derived life times of active substrate binding poses explain KM of laccase mutants. *RSC Adv* **8**:36915–36926.

Miller, B.R., McGee. T.D., Swails, J.M., Homeyer, N., Gohlke, H., and Roitberg, A.E. (2012) MMPBSA.py: An efficient program for end-state free energy calculations. *J Chem Theory Comput* **8**: 3314-3321.

Pettersen, E.F. *et al.* (2004) UCSF Chimera - A visualization system for exploratory research and analysis. *J Comput Chem* **25**: 1605–1612.

Roe, D.R. and Cheatham, T.E. (2013) PTRAJ and CPPTRAJ: Software for processing and analysis of molecular dynamics trajectory data. *J Chem Theory Comput* **9**: 3084–3095.

Waterhouse, A., Bertoni, M., Bienert, S., Studer, G., Tauriello, G., Gumienny, R., Heer, F.T., de Beer, T.A.P., Rempfer, C., Bordoli, L., Lepore, R., and Schwede, T. (2018) SWISS-MODEL: Homology modelling of protein structures and complexes. *Nucleic Acids Res* **46**: W296–W303.

Xu, F. (1997) Effects of redox potential and hydroxide inhibition on the pH activity profile of fungal laccases. *J Biol Chem* **272**: 924–928.
